# Hyoid bone position in different facial skeletal patterns

**DOI:** 10.4317/jced.54657

**Published:** 2018-04-01

**Authors:** Samare Mortazavi, Hamed Asghari-Moghaddam, Mahboobe Dehghani, Mohammadreza Aboutorabzade, Banafshe Yaloodbardan, Elahe Tohidi, Seyed-Hosein Hoseini-Zarch

**Affiliations:** 1 Assistant Professor, Oral and Maxillofacial Radiology Department, Mashhad Dental School, Mashhad University of Medical Sciences, Mashhad, Iran; 2 DDS, Mashhad Dental School, Mashhad University of Medical Sciences, Mashhad, Iran; 3 Assistant Professor of Orthodontics, Dental Research Center, Mashhad University of Medical Sciences, Mashhad, Iran; 4 Students Research Committee, Mashhad University of Medical Sciences, Mashhad, Iran; 5 Associate Professor of Oral and Maxillofacial Radiology, Dental Materials Research Center, Mashhad University of Medical Sciences, Mashhad, Iran

## Abstract

**Background:**

Hyoid bone plays a significant role in physiological functions of craniofacial region and it’s position adapts to changes of the head. The purpose of this study was to determine the position of the hyoid bone among subjects with class I, class II and class III skeletal patterns and evaluate the gender differences.

**Material and Methods:**

One hundred and ten lateral cephalograms (59 females and 51 males) from different skeletal patterns (class I, II and III) were selected. The skeletal patterns were determined according to ANB angle. Using MicroDicom software, different linear and angular measurements (6 variables) was carried out to determine the position of hyoid bone. Intraclass correlation coefficient was used to verify reliability. Descriptive statistics of the variables were calculated and analyzed using two-way ANOVA and Bonferroni statistical methods.

**Results:**

The mean distance from the hyoid bone (H) to mandibular plane (MP), to palatal plane (PP), as well as to a third cervical vertebra (C3) was more in males than females (*p*=0.023, *p*<0.001, *p*<0.001 respectively). The mean H to PP distance was significantly more in skeletal class I compared to class III (*P*=0.01). The mean H to C3 distance was significantly more in skeletal class I compared to class II (*P*=0.008). The mean angle between H-MP and H-PP did not show any statistical difference among three skeletal classes (*p*=0.102, *P*=0.213) and among male and female groups (*P*=0.172, *P*=0.904).

**Conclusions:**

The hyoid bone is positioned more superior and posterior in females than males and its location differs among different skeletal classes. It is placed more posterior in skeletal class II patterns and more inferior and anterior in skeletal class I patterns.

** Key words:**Hyoid bone, Lateral cephalometry, Class III, Class II, skeletal pattern, Orthodontics.

## Introduction

It has been recognized that there may be significant differences in physiologic function of individuals with different craniofacial anatomical relationships (1). In orthodontics, cephalometric radiography has become one of the most essential tools for recognizing the craniofacial anatomical relationships. It has been utilized extensively to quantify the dental, skeletal and soft tissue relationships of the craniofacial complex, prior to the beginning of orthodontic treatment and throughout growth. Less often, in clinical research, cephalometry is used to evaluate cranio-cervical angulation, pharyngeal relationships, soft palate dimensions and hyoid bone and tongue position (2-5).

Throughout the previous two decades, substantial attention has been given to the position of the hyoid bone relative to the facial skeleton (4,6,7). The hyoid bone may be used as an anatomical feature relating the position of the head with the neck. The hyoid bone is connected to the skull base, and on the other hand it is connected to the mandible (8).

This bone plays a significant role in physiological functions such as ingestion, respiration and speaking. Researchers have shown that changes of mandibular position are pertinent to the hyoid bone alterations, and the hyoid bone position adapts to antero-posterior changes of the head (10-12). Also, adaptation of hyoid bone position after orthognathic operations has been revealed (13,14).

During the postnatal period, sexual differences in hyoid bone position appear. Until now, few studies have investigated the gender differences in the position of hyoid bone.

We have found no analytical studies on the position of the hyoid bone in different skeletal patterns and considering gender in Persian ethnic groups. Hence, this study was designed to investigate the position of the hyoid bone in subjects with different antero-posterior skeletal patterns (class I, class II and class III). We also aimed to investigate the gender differences in the hyoid bone position.

## Material and Methods

The research was confirmed by the Ethics Committee of the Research Deputy of Mashhad University of Medical Sciences, Mashhad, Iran (protocol no: 931542).

This research was designed as a cross-sectional study. Lateral cephalometric radiographs as digital images (JPEG format) were selected from patients who were referred to a private oral and maxillofacial radiology center in Mashhad, Iran. All the patients were of Persian ethnic origin.

The following inclusion criteria were considered: 1- Patients with lateral cephalogram taken in the natural head position where at least the fourth cervical vertebrae is depicted 2- minimum age of 18 years 3- complete dentition (except for the third molars) 4- typical facial skeletal pattern I, II, or III. In class II and III groups, preferably sever cases who had significant overjet/reverse overjet and need orthognathic surgery were selected. The exclusion criteria deployed were : 1- severe long or short face 2- significant mandibular rotations 3- previous orthodontic or orthognathic surgery 4- severe respiratory or swallowing disorders 5- history of head and neck trauma, vertebral column and craniofacial anomaly or syndrome 6- presence of radiographic distortions.

An orthodontist evaluated the lateral cephalograms according to the inclusion and exclusion criteria and, 110 lateral cephalograms (59 females and 51 males) were subsequently chosen from 3 classes of anteroposterior skeletal patterns. The sample consisted of 41 subjects with class I, 36 with class II and 33 with class III skeletal patterns.

-Lateral Cephalograms

The lateral cephalograms were taken with the same machine (PlanmecaProMax, Helsinki, Finland) in the natural head position with the Frankfurt plane parallel to the floor as standard (15). The ear rods of the radiography apparatus were not positioned inside the hearing conduits, but after determination of natural head position they were placed gently close to the external part of the auditory meatus to stabilize the head posture during exposure. The distance from the focus to the median plane was 150 cm and from the median plane to the film was 15 cm. As a result, the enlargement was 10 percent. All subjects were asked not to swallow, breathe through their nose and to contact their teeth lightly while the radiographs were being taken. Subjects were further instructed to have their lips in light contact, position tip of their tongue behind the maxillary central incisors with teeth in occlusion.

-Cephalometric Analysis

Linear and angular measurements were done using MicroDicom software (version 0.8.8). All measurements were carried out by the same radiologist. The sagittal skeletal pattern of subjects (Class I, Class II or Class III) was determined by measuring the ANB angle, under an orthodontist’s supervision. The classifications based on ANB angles were as follow: Class I (ANB angle = 0-4), class II (ANB angle > 4) and Class III (ANB angle < 0).

A total of 6 variables were evaluated on the lateral cephalograms. The definition of the measurement points, lines and reference planes on the lateral cephalograms used in this study which have been described in previous studies, (9,16) are presented in  Table 1.

**Table 1:**
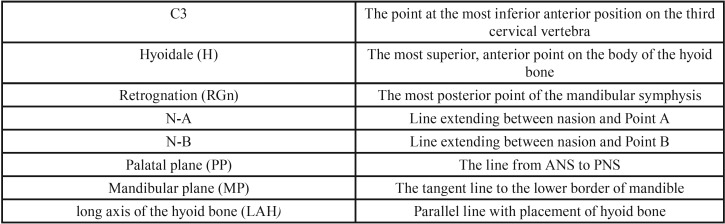
Definition of the measurement points, lines and reference planes on the lateral cephalometric radiographs used in this study.

**Figure 1:**
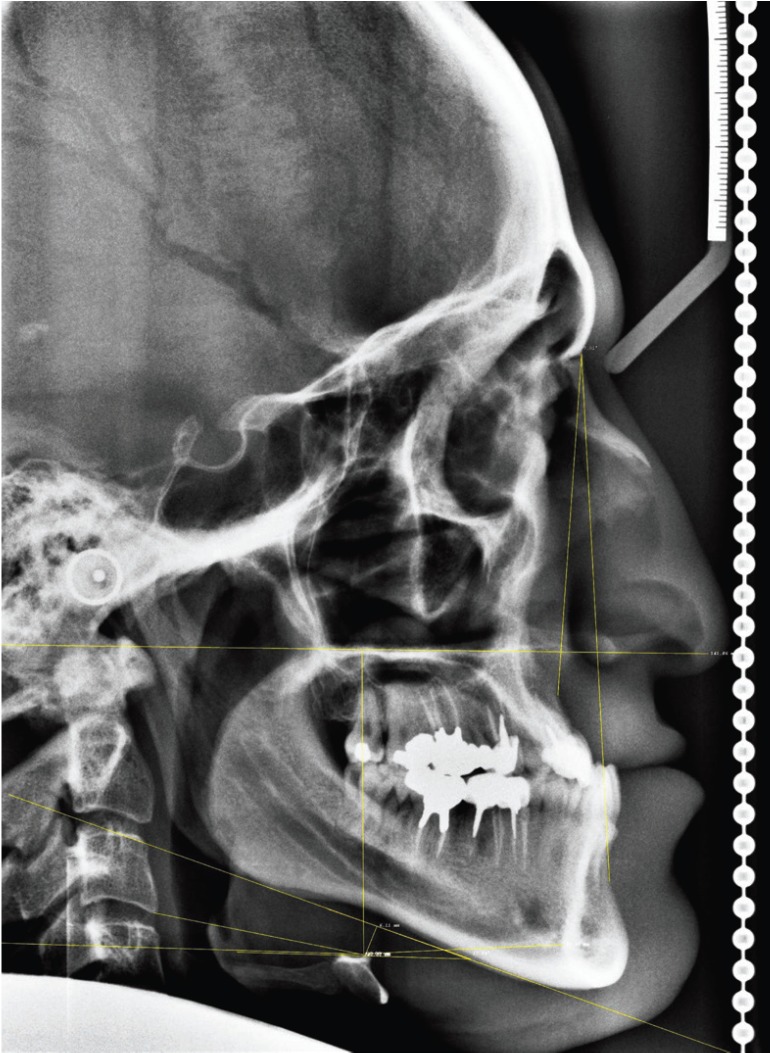
Linear and angular measurements of hyoid bone position on the lateral cephalometric radiograph used in the study.

The linear and angular (Fig. 1) measurements made were:

• H-MP: Perpendicular distance from the most superior-anterior point on the body of the hyoid bone (Hyoidal: H) to the mandibular plane (MP).

• H-PP: Perpendicular distance from the hyoidal to the palatal plane (PP).

• H-C3: Distance from the hyoidal to the most inferior anterior point on the third cervical vertebra (C3).

• H‑RGn: Distance from the hyoidal to the most dorsal point of the mandibular symphysis (Retro-gnathion (RGn)).

• LAH‑MP: Angle formed by the long axis of the hyoid bone (LAH) and the mandibular plane (MP).

• LAH-PP: Angle formed by the long axis of the hyoid bone (LAH) and the palatal plane (PP).

Following the measurements, the relationships between hyoid bone position and skeletal pattern and gender were evaluated.

-Reliability

Two weeks after the first measurements, 15 radiographs were selected randomly and re-measured by the same radiologist and intra-class correlation coefficient (ICC) between first and second measurements was calculated.

-Statistics

Statistical analyses were performed using SPSS software package (version 11.5). Descriptive statistics (mean and standard deviation (SD)) were calculated for each measurement. The statistical tests used were Shapiro-Wilk, two-way ANOVA, and Bonferroni tests. *P*<0.05 was considered as statistically significant.

## Results

There were a total of 59 females (53.6%) and 51 males (46.4%). Of the 110 subjects there were 41 with a class I (37%), 36 with a class II (33%) and 33 with a class III skeletal pattern (30%).

The ICC values for the measurements ranged from 0.94 to 0.99 demonstrating excellent reliability.

-H-MP:

The mean distance from the H point to mandibular plane was significantly more in males than females, as it is clearly demonstrated in  Table 2 (*p*=0.023).

**Table 2:**
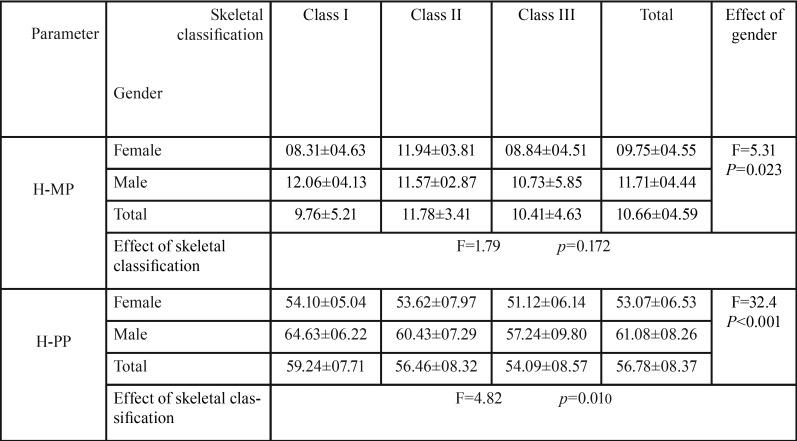
Mean and SD of vertical linear parameters (H-MP, H-PP) in different skeletal classifications based on gender.

The mean distance from the H point to mandibular plane was lowest for skeletal class I and highest for skeletal class II ( Table 2).

-H-PP:

As shown in  Table 2, the mean distance from the H point to palatal plane was found to be significantly more in males compared to females (*p*<0.001).

The mean H-PP distance was least in skeletal class III and highest in skeletal class I. Using Bonferroni test, it was revealed that the value for skeletal class I is significantly more than skeletal class III ( Table 2).

-H-C3:

The mean distance from H point to C3 was significantly more in male subjects compared to females ( Table 3) (*p*<0.001).

**Table 3:**
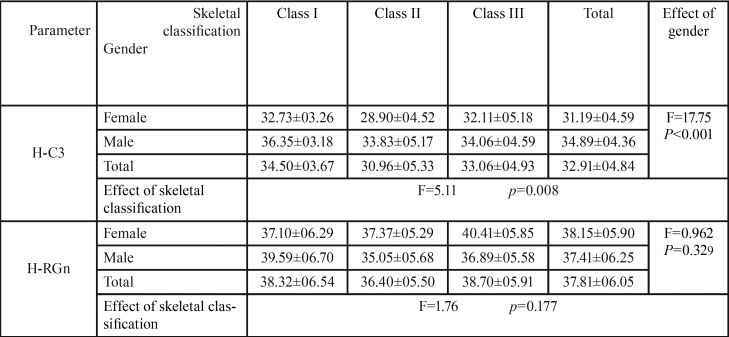
Mean and SD of horizontal linear parameters (H-C3, H-RGn) in different skeletal classifications based on gender.

The skeletal class II subjects had the lowest mean H-C3 distance whilst this distance was the highest in skeletal class I subjects, and a meaningful difference was detected among three skeletal classes (*p*=0.008). Performing pair wise comparison using Bonferroni test, the difference between skeletal class I and II was only found to be statistically significant ( Table 3).

-H-RGn:

As seen in  Table 3, the mean distance from the H point to RGn was less in males than females, but this difference was not found to be statistically significant (*p*=0.329).

The mean H-RGn distance was least in skeletal class II cases and most in class III, however, no significant difference was observed among the three skeletal classes (*p*=0.177) ( Table 3).

-LAH-MP:

The mean angle between the hyoid bone and mandibular plane was higher in males than females, but this difference was not statistically significant (*p*=0.172). Class I and Class III subjects has the lowest and highest mean LAH-MP angles respectively. However, there was no statistical difference among the three skeletal classes (*p*=0.102) ( Table 4).

**Table 4:**
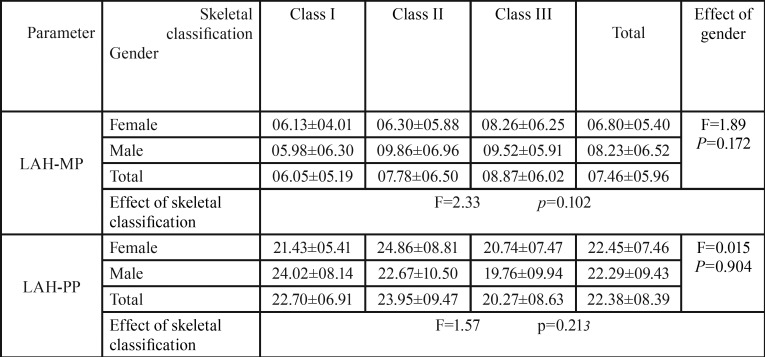
Mean and SD of angular parameters (LAH-MP, LAH-PP) in different skeletal classifications based on gender.

-LAH-PP:

As indicated in  Table 4, the mean angle between the hyoid bone and palatal plane was lower in males compared to females, however, this difference was not statistically significant (*p*=0.904).

The mean LAH-PP angle was lowest for skeletal class III and highest among class II cases, but this difference among three skeletal classes was not found to be statistically significant (*p*=0.213) ( Table 4).

## Discussion

In the early stages of life, the hyoid bone is placed at the inferior edge of mandibular border, but with the aging process, it gradually descends and eventually is fixed next to the fourth cervical vertebra (C4). The hyoid bone is unique in not having joints with any other bone and floating in connection with ligaments and muscles. Thus, the position of hyoid bone differs as a result of any changes in the body gesture, head position and other physiological states. In addition, the hyoid bone moves in response to mouth’s different functions such as respiration and ingestion.

Extensive research has been conducted in order to identify the position of the hyoid bone in various dentofacial patterns. The studies have shown that changes in the position of the mandible are related to those in the hyoid bone and the position of hyoid bone adjusts to antero-posterior changes in the head posture (13,14). Moreover, published studies have determined that there is a strong relation between the hyoid bone position and the size of respiratory tract, therefore, great consideration has to be given to this prior to orthognathic surgery (13,17-19).

In the present study it has been shown that the hyoid bone was placed inferior in males compared to females for all subjects in three classes of skeletal pattern. This is in agreement with the result of the study by Sahin Saglam (20) which, concluded that the hyoid bone position is superior and posterior in females.

In the current investigation the distance from the H point to the third cervical vertebra (C3) is longer in males than females, which is contrary to the previous studies (16,21). Considering the larger size of muscles, bones, and overall skeleton in males, it is expected that the distance from hyoid bone to adjacent landmarks be more in males than females, which is in accordance with this study’s findings. On the other hands, smaller distances in females from the hyoid bone to craniofacial structures is simply due to the difference in average gender size.

In this research, it was observed that the distance from H point to palatal plane (H-PP) was greatest in class I subjects and the least in class III subjects and this difference was statistically significant (*P*=0.01). In contrast to findings of our study, Allhaija *et al.* concluded that in skeletal class II patients, vertical position of the hyoid bone was significantly closer to the mandible in comparison with the other two groups (21). The difference between the results of our study and the other study may be due to racial differences.

The distance between H point and third cervical vertebra (C3) in this investigation was the least in skeletal class II and the most among class I subjects. This reflects that the hyoid bone is positioned more anteriorly in class I patients with Persian ethnic background. This was in keeping with a previous study by Allhaija *et al.* (21), which showed that the H-C3 distance in class II patients was significantly less compared to those with class I and III skeletal pattern. Nevertheless, in the study by Jose et al in 2015, it was concluded that the position of hyoid bone in antero-posterior dimensions does not have any statistical difference among individuals with skeletal I, II and III patterns (22). The findings of a different study by Tekale is in agreement with our results where, the position of the hyoid bone in horizontal dimension is closer to cervical vertebras in skeletal class II patients.

According to our results ( Table 3), the horizontal distances from the H point to the adjacent landmarks (third cervical vertebra and retrognathion) were less in class II subjects compared to other skeletal classes. This may be reflective of the smaller mandibular size and retruded position of mandible in skeletal class II subjects.

In the present study, the difference in distance from the H point to RGn was not statistically significant among three skeletal classes and also among males and females, although it was slightly smaller in class II subjects and in males.

The results of the angular measurements of hyoid bone failed to detect any significant differences among the three skeletal patterns. This was in agreement with results of a study by, Chauhan *et al.* which reported no difference in angular measurements of hyoid bone between class I and class II Div 1 subjects (10).

A limitation of current study was the absence of functional assessment of hyoid bone. Therefore, we suggest for the position of hyoid bone to be investigated during functions such as swallowing, speach and breathing in future research.

As conclusion of current study, the hyoid bone is positioned more superior and posterior in females compared to males in the studied subjects of Persian ethnicity. The location of hyoid bone differs among different skeletal classes. The horizontal distance of hyoid bone to adjacent landmarks (third cervical vertebra and retrognathion) was less in class II subjects compared to the other skeletal classes, indicating that the hyoid bone is placed more posterior in skeletal class II pattern. Moreover, the hyoid bone is positioned more inferior and anterior in skeletal class I pattern. The angular measurements of the hyoid bone does not show any significant difference among three skeletal classes.
